# Pedicle Screw Fixation Study in Immature Porcine Spines to Improve Pullout Resistance during Animal Testing

**DOI:** 10.1371/journal.pone.0127463

**Published:** 2015-10-09

**Authors:** Sophie Le Cann, Thibaut Cachon, Eric Viguier, Lotfi Miladi, Thierry Odent, Jean-Marie Rossi, Patrick Chabrand

**Affiliations:** 1 Aix-Marseille Université, CNRS, ISM UMR 7287, 13288, Marseille cedex 09, France; 2 EUROS, Z.E. Athélia III, 824 Voie Antiope, 13600, La Ciotat, France; 3 APHM, Hôpital Sainte-Marguerite, Institute for Locomotion, 13009, Marseille, France; 4 ICE UPSP, VetAgro Sup, Campus vétérinaire de Lyon, University of Lyon, F-69280, Marcy l'Étoile, France; 5 Hôpital Universitaire Necker Enfants malades, Assistance Publique Hôpitaux de Paris, Faculté de médecine Paris Descartes, Université Sorbonne Paris Cité, Paris, France; Faculty of Animal Sciences and Food Engineering, University of São Paulo, BRAZIL

## Abstract

The porcine model is frequently used during development and validation of new spinal devices, because of its likeness to the human spine. These spinal devices are frequently composed of pedicle screws with a reputation for stable fixation but which can suffer pullouts during preclinical implantation on young animals, leading to high morbidity. With a view to identifying the best choices to optimize pedicle screw fixation in the porcine model, this study evaluates ex vivo the impact of weight (age) of the animal, the level of the vertebrae (lumbar or thoracic) and the type of screw anchorage (mono- or bi-cortical) on pedicle screw pullouts. Among the 80 pig vertebrae (90- and 140-day-old) tested in this study, the average screw pullout forces ranged between 419.9N and 1341.2N. In addition, statistical differences were found between test groups, pointing out the influence of the three parameters stated above. We found that the the more caudally the screws are positioned (lumbar level), the greater their pullout resistance is, moreover, screw stability increases with the age, and finally, the screws implanted with a mono-cortical anchorage sustained lower pullout forces than those implanted with a bi-cortical anchorage. We conclude that the best anchorage can be obtained with older animals, using a lumbar fixation and long screws traversing the vertebra and inducing bi-cortical anchorage. In very young animals, pedicle screw fixations need to be bi-cortical and more numerous to prevent pullout.

## Introduction

New medical devices require not only biocompatibility but also “biofunctionality”, with a proof of concept. Thus, the pre-clinical phase of development frequently involves the use of animal experimentation. However, this raises the familiar issue of the pain and distress caused to the animals used for research. The “Three Rs” ethical concept, standing for Replacement, Reduction and Refinement, was first described by Russel and Burch in 1959 [1] and aims to protect animals through useful and appropriate experimentation. The porcine model is commonly used to validate spinal implants [2,3] because it most closely matches the pediatric spine in terms of size and shape of vertebrae [4]. Pedicle screws are widely accepted as an anchorage system in many surgical vertebral stabilization systems for their safety and stability. Furthermore they have several advantages over other fixation methods [5–7]. Traversing all three columns of the vertebrae, they can rigidly stabilize both the ventral and dorsal aspects of the spine. Pedicle screw fixation does not require intact dorsal elements; it can be used after a laminectomy or traumatic disruption of lamina, spinous processes and/or facets and does not violate the vertebral canal when inserted correctly. Used in conjunction with metallic systems, pedicle screws enhance fusion and decrease the number of vertebral fixations along the spine compared to other fixation systems (hooks for example) [7]. The pedicle represents the strongest point of attachment of the vertebra, and allows significant multi-planar forces to be applied to the spine through pedicle screws without failure of the bone-metal junction.

Pedicle screws are widely used in treating spinal diseases such as scoliosis [6–9] and other vertebral pathologies [10–12]. In animal research, pedicle screws are commonly used in scoliosis models aimed at developing human therapeutic treatments [13,14]. Creating scoliosis in the porcine model has been achieved in the past [13,15–18] through the use of flexible tethers or stainless steel cables. The anchorage for a pedicle screw is commonly mono-cortical, with an ideal screw penetration of around 70% of the vertebral body [6]. However, to enhance stability for animal experiments, bi-cortical anchorage may be used with long screws traversing the vertebra. Although this anchorage was tested on pigs without any resulting vascular issues [17], during previous animal studies the veterinary community noted screw pullouts. Those observations may be explained by the soft bone of young animals [16,17], small vertebrae size, and high loads sustained by the screws; forces above 750N have recently been observed during the creation of scoliotic deformities [19]. Fixation of pedicle screws depends on various parameters such as bone quality and screw-bone interface [20]; if pedicle screws are not well-anchored, animals may sustain more pain. At worst, pullouts could induce animal death, and the ensuing need for additional animals has obvious ethical as well as financial implications for developmental models. In the interests of efficiency, therefore, better knowledge of the resistance of the screws used in animal models for spinal device development is vital, so as to keep morbidity to a minimum.

Although the pedicle fixation technique is not new, the literature contains little information on the pullout resistance of pedicle screws inserted in young pigs. Existing studies evaluate the impacts either of screw design [21] or of insertion technique [4], or of type of anchorage system (screws or hooks [22]) on pedicle screw stability. It is important to study and take into account new parameters that could influence pedicle screw stability. To the authors’ knowledge, no comparison has been made of the pullout resistance of pedicle screws inserted in porcine spines related to age of the animal, level of the vertebrae or type of anchorage of the screws. Yet this information would clearly help determine the best options for implantation of pedicle screws.

The objective of this study is to evaluate pedicle screw fixation and thereby identify the most relevant parameters to be optimized in future experimentation on this porcine model. To do so, we assessed the influence of 1) the level of the vertebrae (lumbar and thoracic), 2) the weight of the animals (30kg and 50kg, which corresponds to an age of 90 and 140 days) and 3) the cortical anchorage (mono- and bi-cortical) on the ultimate pedicle screw pullout forces.

## Materials and Methods

### Sample preparation

We chose as animal model the landrace pig, known to most closely approximate the size and shape of human vertebrae [22]. Eight immature cadaveric porcine spines were collected following previous studies not affecting their spines: 4 of 30kg (approximate age 90 days) and 4 of 50kg (approximate age 140 days) at the Institut Claude Bourgelat in Lyon. We were not the actors of those previous in vivo studies; they were realized at the Institut Claude Bourgelat, and approved by the National Institutes of Health, with ethic projects number 1065 and 1341 (Institutional Study Committee approved by French Education and Research Ministry). This guarantees that the previous euthanasias were carried out in strict accordance with the recommendations in the Guide for the Care and Use of Laboratory Animals. From each spine, 5 lumbar (L1–L6) and 5 thoracic (T5–T9) vertebrae were extracted. All 80 vertebrae were frozen at -20°C immediately after extraction.

**Fig 1:**
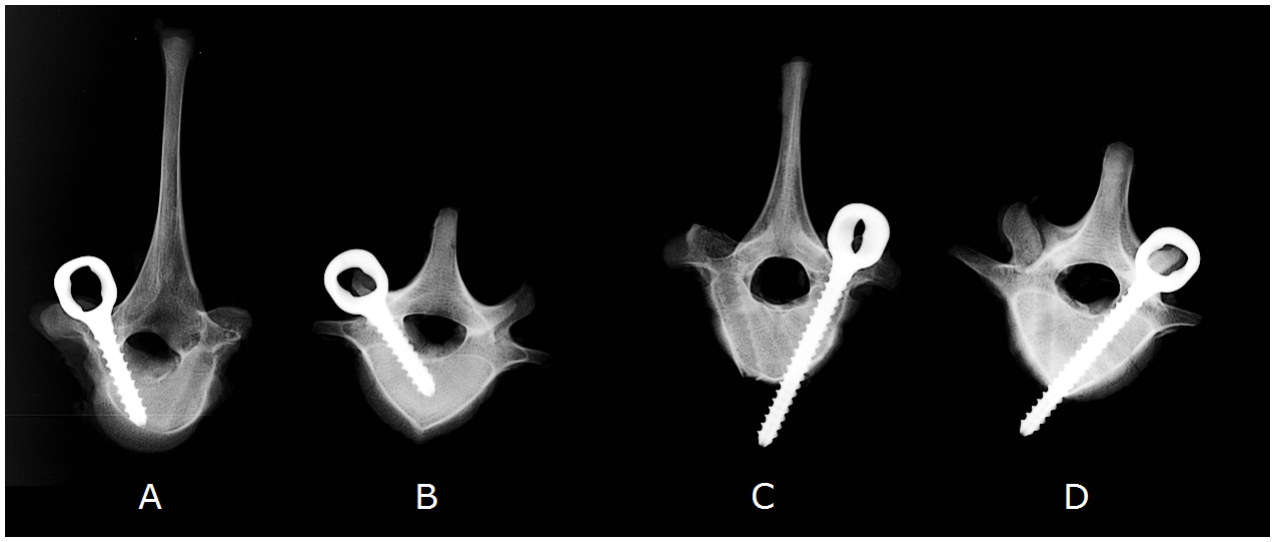
X-rays of thoracic (A, C) and lumbar (B, D) vertebrae of a 90-day-old pig (30kg), with implanted screws 20mm (A, B) and 40mm (C, D) long.

The pedicle dimensions of the animal model at these ages are given in Table 1 (anatomical measurements, Osirix Imaging Software). We based our choices of screw dimensions on a review by Suk, advising a maximum of 80% of the pedicle diameter for the screw diameter [6], which here limits the diameter to 6 mm. The pedicle screws tested (EUROS SAS, La Ciotat, France) are therefore 4.35 mm diameter, 1.95 mm thread pitch and 0.6 mm thread depth, based on existing designs. Two screw lengths were used for this study so as to induce two different cortical anchorages: mono- and bi-cortical. For the first anchorage, the standard mono-cortical pedicle insertion, Suk advises an ideal length of 70% penetration of the vertebral body [6], which here represents a range between 19.9 mm and 21.4 mm (Table 1); we used 20 mm long screws (Fig 1A and 1B). For the bi-cortical anchorage, we used 40 mm long screws to ensure that they traversed the vertebral body, crossing the anterior cortical part of the vertebral body on a perpendicular line (Fig 1C and 1D).

**Table 1:** Pedicle dimensions for porcine vertebrae according to weight (age) of animal, vertebra level and type of measurements.

	Thoracic region (T5–T9)		Lumbar region (L1–L5)	
	Pedicle width	Length	Pedicle width	Length
30kg pigs (90 days)	7.6±0.3 mm	28.7±3.1 mm	8.7±0.3 mm	28.5±0.6 mm
50kg pigs (140 days)	8±0.4 mm	30.6±1.9 mm	9.1±0.4 mm	29.8±2 mm

Length is sum of pedicle and vertebral body lengths. Values are mean ± SD, averaged from 50 measurements.

All pedicle screw insertions were performed with a free-hand technique, with a convergent direction (Magerl insertion technique [23]) by the same senior veterinary surgeon. The entry point was chosen at the cross section between the bottom of the articular process and a horizontal line drawn in the middle of the transverse process [24]. After determination of this entry point, the cortical was perforated with a small awl. The hole was then prepared with a pedicle finder, with no pre-tapping. Each screw was gently inserted with a progressive insertional torque until immediate cortical contact of the screw head to avoid micro fracture of bone cortex. X-rays were performed after insertion to assess the position of the screws (Fig 1). No vertebra was excluded due to malposition (no medial cortical wall violation or breach in the foramen was observed).

Each vertebra was then casted in polyurethane resin (F1, Axson) in a PVC circular container 100mm in diameter and between 25mm and 35mm high, depending on the size of the vertebra. The heating associated with the hardening of this particular resin was previously proved not to degrade bone [25]. Playdough was used to cover the tips of the screws (for the bi-cortical configuration), to avoid contact between the screw and the resin, and was also placed inside the vertebral canal. The vertical alignment of the screws was checked during resin hardening. Each embedded vertebra was then inspected to ensure that the resin did not interfere with the screw.

### Pullout tests

**Fig 2:**
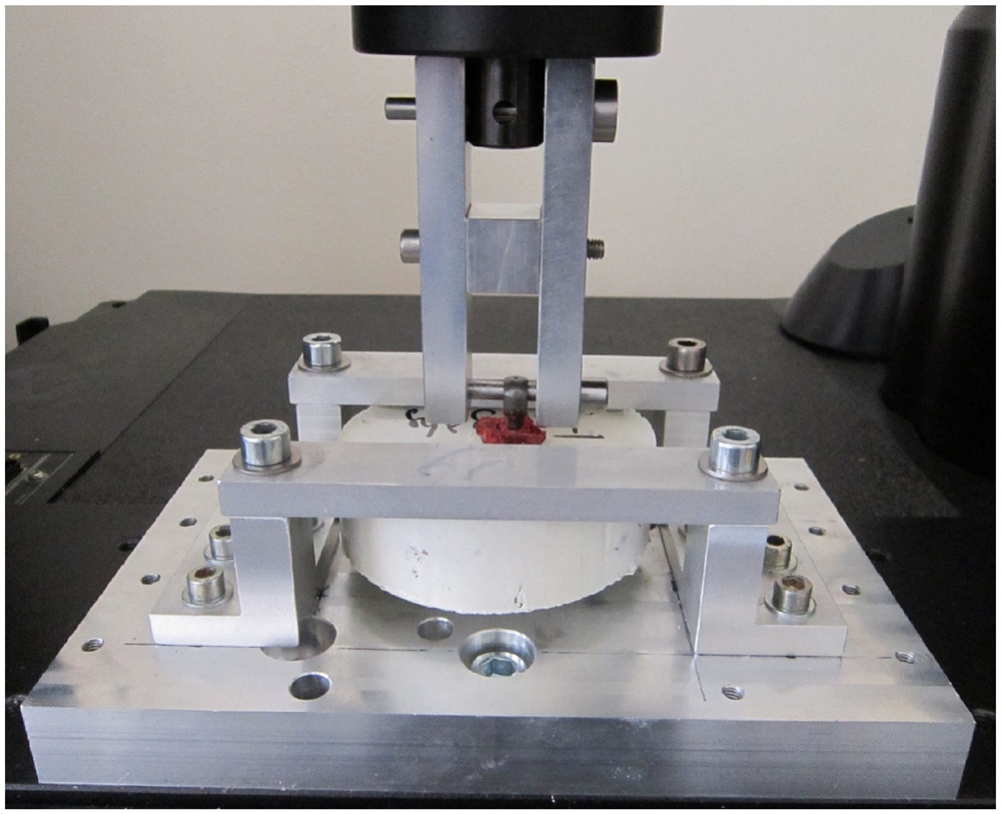
Pullout assembly.

The screws were tested on a mechanical testing machine (MTS INSTRON 5566A), using a load cell with maximum capacity 10 kN and precision ±0.5%. A special assembly was developed to firmly maintain the embedded vertebra with kind of metallic shelf brackets (see Fig 2). The head screw was attached to the load cell of the testing machine. A preload of 10 N was applied to set up the system and then a constant ascendant displacement speed of 3mm/min was applied in line with the screw axis until the screws were extracted. We monitored forces *vs* displacements during the tests, and noted the maximum pullout force for each tested vertebra.

Mean values and standard deviations (SD) were calculated for each set of 10 repeats. A total of 8 different configurations of type of anchorage, vertebra level and weight of animal were tested. A non-parametric statistical study was realized on the results in pairs, using the Mann Whitney test.

## Results

During the tests, two vertebrae were eliminated because of poor resin casting (the vertebra slipped), in the 50kg, bi-cortical, lumbar configuration. At the end of the tests, 78 vertebrae (thus 78 screws) were available for analysis. Pullout forces were averaged from the ten repeats for each level (all thoracic and all lumbar vertebrae), weight and type of anchorage; results are shown in Table 2. The average pullout forces range between 419.9 and 1341.2N.

**Table 2:** Pullout forces averaged for 10 repeats.

Pig weight		30kg (approximate age 90 days)		50kg (approximate age 140 days)	
Vertebra level		Lumbar	Thoracic	Lumbar	Thoracic
Mono-cortical	Average pullout forces (in N)	761.1±55.1	419.9±105.8	1076.7±84	594.3±106.7
Bi-cortical	Average pullout forces (in N)	993±126.2	682.2±127.7	1341.2±199.6ª	954.2±180.4

Values are mean ± SD.

^a^ Only 8 repeats.

Results of the statistical tests on the pullout forces are presented in Table 3 through p-values. Statistical differences were found between most tested groups; only 5 comparisons returned non-statistical differences, which are in italic font in Table 3. When we compared young and aged animals, we found statistical differences on the pullout forces between 30kg’s and 50kg’s animals (4 groups, p<.01). Concerning the vertebral level, statistical differences were also found between lumbar and thoracic vertebrae (4 groups, p<.01) as well as for the type of anchorage, between mono- and bi-cortical implantation (4 groups, p<.01).

**Table 3:** P-values for pullout forces from Mann Whitney statistical test.

p-values	Mono-T 50kg	Mono-L 30kg	Mono-L 50kg	Bi-T 30kg	Bi-T 50kg	Bi-L 30kg	Bi-L 50kg
Mono- T 30kg	.006**	<.0001**	<.0001**	.001**	<.0001**	<.0001**	<.0001**
Mono- T 50kg		.001**	<.0001**	.*121*	.001**	<.0001**	<.0001**
Mono- L 30kg			<.0001**	.*273*	.014*	.001**	<.0001**
Mono- L 50kg				<.0001**	.*162*	.*140*	.002**
Bi- T 30kg					.003**	.001**	<.0001**
Bi- T 50kg						.*678*	.002**
Bi- L 30kg							.001**

* Means p <.05

** p <.01.

In italic font, non-statistically different results (p>.05). Mono- and Bi- stand for Mono-cortical and Bi-cortical anchorage. T and L stand for Thoracic and Lumbar level.

**Fig 3:**
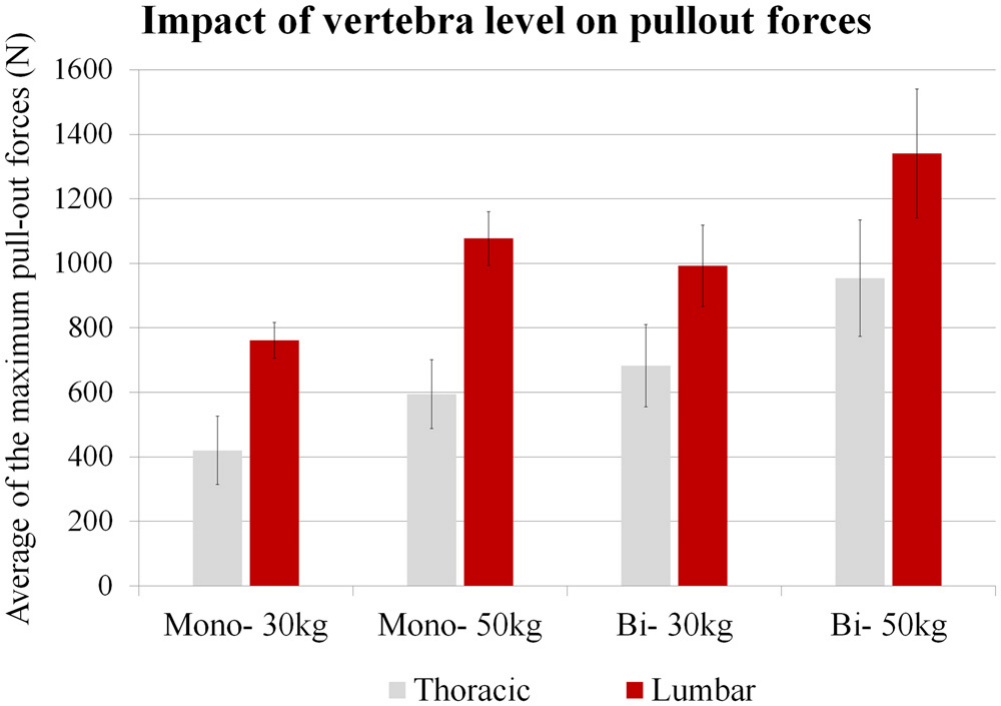
Impact of vertebra level, lumbar or thoracic, on the averaged pullout forces. Mono- and Bi- respectively stand for Mono-cortical and Bi-cortical anchorage of the screws.

**Fig 4:**
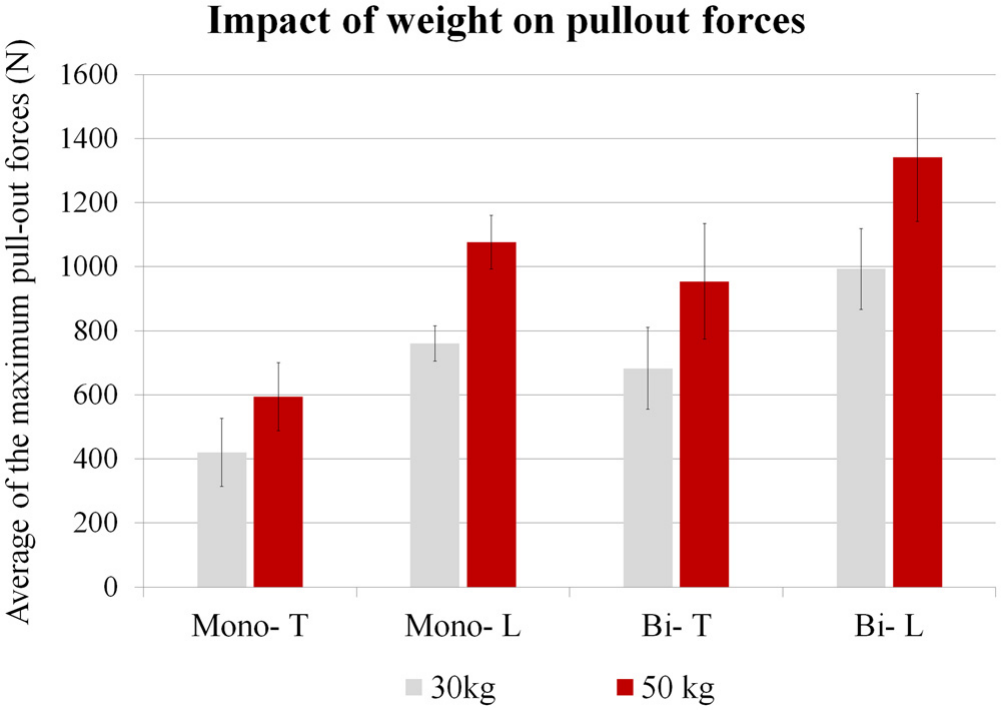
Impact of animal’s weight: 30kg (90 days) or 50kg (140 days) on the averaged pullout forces. Mono- and Bi- respectively stand for Mono-cortical and Bi-cortical anchorage of the screws. T and L stand for Thoracic and Lumbar vertebrae.

**Fig 5:**
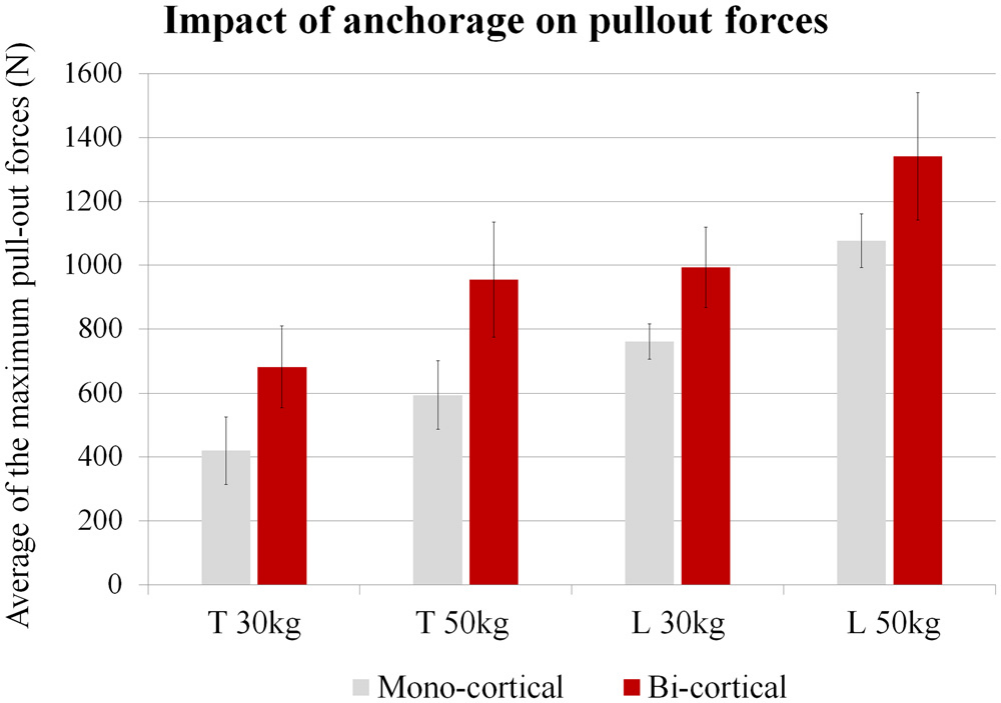
Impact of type of screw anchorage, mono- or bi-cortical, on the averaged pullout forces. T and L stand for Thoracic and Lumbar vertebrae.

The following three graphs highlight the respective influence of vertebra level (Fig 3), weight (age) (Fig 4) and type of anchorage (Fig 5) on the averaged pullout forces. For both types of anchorage (mono- and bi-cortical) and for each weight we found that thoracic vertebrae sustained significantly lower pullout forces than lumbar vertebrae (p<.01). For both levels (thoracic and lumbar), the vertebrae of the 30kg pigs were found to sustain lower pull-out forces than those of the 50kg pigs (p<.01). When types of anchorage are compared, for any weight and vertebra level the mono-cortical anchorage induced lower pullout forces than the bi-cortical anchorage (p <.01).

## Discussion

Current medical device development processes frequently require an experimental phase using animals, to assess efficacy before implantation in humans. During previous studies on the pig model to validate spinal devices, some veterinarians and researchers noted screw pullouts [16,17] leading to high animal morbidity. In line with the ethical concept of the “Three Rs”, this study investigated how certain parameters (pig weight (age), vertebra level and type of cortical anchorage) impact pedicle fixation in a young porcine model, in order to optimize pedicle fixation. We observed the highest pullout forces for the lumbar level, the bi-cortical anchorage and the oldest animals.

Yazici et al [4] studied the effect of dilatation of the pedicles on screw stability using two-month-old pigs, and found a mean pullout force of 408.1±102N for non-dilated pedicles (thoracic and lumbar vertebrae, with screws of respectively Ø 3.5 mm and Ø 4 mm). Abshire et al [21] studied the impact of the geometry of the screws (conical vs cylindrical, respectively Ø 7.5 mm and Ø 6.5 mm, and 40 mm long), but using mature pigs (70kg–90kg); they found a mean pullout force of 2634.1N for lumbar vertebrae. Our study using three- and five-month-old pigs (30kg and 50kg) yields values consistent with the literature: higher than for 2-month-old pigs and lower than for pigs weighing 70–90kg. These are the first average values for ultimate pullout forces of pedicle screws on such young animals, ranging between 420N and 1340N.

When we investigated how the level of the vertebra affects screw pullout forces, the results for a given weight and anchorage were statistically different between the thoracic and lumbar level (p<.01) (Fig 3). We found that the more caudally the screws are positioned (lumbar level), the greater their pullout resistance is, with an increase of approximately 80% for mono-cortical anchorage and 45% for bi-cortical anchorage. This could be explained by the size of the vertebrae: lumbar vertebrae are wider than thoracic, especially in terms of pedicle dimensions (Table 1): lumbar pedicle width is around 14% higher than thoracic at 30kg, and 13% higher at 50kg.

Looking at how animal weight (age) of the animals affects pedicle screw pullout resistance, results for a given level and anchorage were statistically different between 30kg and 50kg (p<.01) (Fig 4). Screw stability increases with the age of the animals. Between 3 and 5 months of age the maximum pullout values for the lumbar and thoracic vertebrae increased respectively by 32% and 78% for bi-cortical anchorage and by 52% and 39% for mono-cortical anchorage. This may be explained by the increasing bone mineral density of the vertebrae as pigs grow. It has been reported to rise by around 10% in 2 months of growth [26]; bone is frequently found to be softer by veterinary surgeons implanting screws in very young animals. Moreover, growth also affects the dimensions of the vertebrae (Table 1), increasing both the length of the pedicles (5% to 7% increase in 2 months) and their width (around 5% to 6% increase). These vertebra modifications could explain the increase in primary stability of the screws observed between 90 and 140 days.

Examining the impact of type of cortical anchorage, we obtained results that, for a given age and vertebra level, were statistically different between mono- and bi-cortical anchorage (p<.01) (Fig 5). The screws implanted with a mono-cortical anchorage sustained lower pullout forces than those implanted with a bi-cortical anchorage. Costa et al [27] studied the impact of misalignment of pedicle screws on their pullout resistance in lumbar porcine spines (age of the animals not provided), with different degrees of cortical violation (superior, inferior, medial and lateral). For standard insertions, they obtained forces around 1400N, while cortical violation reduced forces to as low as 600N. In our study, pullout force increased when a second cortical anchorage (anterior aspect of the vertebral body) was added. For the lumbar vertebrae, the increase was 25% and 30% (respectively for 50kg and 30kg), and for the thoracic vertebrae it was 60% and 62% (respectively for 50kg and 30kg). However, the main difference is the orientation of the cortical bone relative to the screw axis. In our study, bi-cortical insertion induces a fairly perpendicular crossing of the anterior cortical part of the vertebral body. When screws are misaligned, the cortical crossing is more tilted and the axis of the screws is not perpendicular to the cortical bone. This underlines the importance for screw stability of whether screws are anchored to one or to two cortical bones, but even more importantly, of the position of the screw crossing the cortical bone. Any bi-cortical insertion must be correctly performed to ensure a cortical crossing perpendicular to the screw axis, thus enhancing the pullout resistance of the screw threads.

This study sought the most relevant parameters for pedicle fixation in a porcine model being used to develop new human implant systems. We found statistical evidence of the impact of age of pigs, level of the implanted vertebra and type of screw anchorage. It would be interesting to extend these tests to osteointegrated screws, to assess the influence of bone healing on their stability. Previous work on the creation of scoliotic deformities suggests that the conservation of an osteointegration period before applying stresses to screws limits the risk of pullouts [17].

One limitation of our study is that pure axial loading does not faithfully represent the in vivo loading sustained by screws used in spinal systems. However, axial loading is commonly used to evaluate screw stability [28]. Moreover, the use of long screws to induce a bi-cortical anchorage may be limited by the risk of vascular issues during the insertion. We only tested here one design of pedicle screw; results may be different with other types of pedicle screws. Other methods could also be used to enhance pedicle fixations such as specific screws [21,29] or augmentation techniques [4].

## Conclusions

To limit screw pullouts, we conclude that the best choices are implantation in older animals, using a lumbar fixation and long screws traversing the vertebra and inducing bi-cortical anchorage. However, bi-cortical anchorage implies that the screw goes beyond the anterior aspect of the vertebral body, and has to be performed with care to avoid any vascular issue. If very young animals are required, pedicle screw fixations need to be bi-cortical to enhance stability, and as many screws as possible should be implanted to prevent avulsion.
